# Neurocognitive Disorders and Dehydration in Older Patients: Clinical Experience Supports the Hydromolecular Hypothesis of Dementia

**DOI:** 10.3390/nu10050562

**Published:** 2018-05-03

**Authors:** Michele Lauriola, Antonio Mangiacotti, Grazia D’Onofrio, Leandro Cascavilla, Francesco Paris, Giulia Paroni, Davide Seripa, Antonio Greco, Daniele Sancarlo

**Affiliations:** Complex Structure of Geriatrics, Department of Medical Sciences, IRCCS “Casa Sollievo della Sofferenza”, San Giovanni Rotondo, Viale Cappuccini 1, 71013 Foggia, Italy; m.lauriola@operapadrepio.it (M.L.); a.mangiacotti@operapadrepio.it (A.M.); l.cascavilla@operapadrepio.it (L.C.); francesco.f.paris@virgilio.it (F.P.); giulia.paroni@operapadrepio.it (G.P.); a.greco@operapadrepio.it (A.G.); d.sancarlo@operapadrepio.it (D.S.)

**Keywords:** hydromolecular hypothesis, dehydration, older patients, neurocognitive disorders

## Abstract

Abnormalities of water homeostasis can be early expressions of neuronal dysfunction, brain atrophy, chronic cerebrovasculopathy and neurodegenerative disease. The aim of this study was to analyze the serum osmolality of subjects with cognitive impairment. One thousand and ninety-one consecutive patients attending the Alzheimer’s Evaluation Unit were evaluated with the Mini-Mental State Examination (MMSE), 21-Item Hamilton Depression Rating Scale (HDRS-21), Activities of Daily Living (ADL), Instrumental-ADL (IADL), Mini Nutritional Assessment (MNA), Exton-Smith Scale (ESS), and Cumulative Illness Rating Scale (CIRS). For each patient, the equation for serum osmolality developed by Khajuria and Krahn was applied. Five hundred and seventy-one patients had cognitive decline and/or depression mood (CD-DM) and 520 did not have CD-DM (control group). Patients with CD-DM were less likely to be male (*p* < 0.001), and were more likely to be older (*p* < 0.001), have a significant clear cognitive impairment (MMSE: *p* < 0.001), show the presence of a depressive mood (HDRS-21: *p* < 0.001) and have major impairments in ADL (*p* < 0.001), IADL (*p* < 0.001), MNA (*p* < 0.001), and ESS (*p* < 0.001), compared to the control group. CD-DM patients had a higher electrolyte concentration (Na^+^: *p* < 0.001; K^+^: *p* < 0.001; Cl^−^: *p* < 0.001), risk of dehydration (osmolality *p* < 0.001), and kidney damage (eGFR: *p* = 0.021), than the control group. Alzheimer’s disease (AD) patients showed a major risk for current dehydration (*p* ≤ 0.001), and dehydration was associated with the risk of developing a type of dementia, like AD or vascular dementia (VaD) (OR = 2.016, *p* < 0.001). In the multivariate analysis, the presence of dehydration state was associated with ADL (*p* < 0.001) and IADL (*p* < 0.001), but independently associated with age (*r*^2^ = 0.0046, *p* = 0.77), ESS (*r*^2^ = 0.0052, *p* = 0.54) and MNA (*r*^2^ = 0.0004, *p* = 0.48). Moreover, younger patients with dementia were significantly more dehydrated than patients without dementia (65–75 years, *p* = 0.001; 76–85 years, *p* = 0.001; ≥86 years, *p* = 0.293). The hydromolecular hypothesis intends to explain the relationship between dehydration and cognitive impairment in older patients as the result of protein misfolding and aggregation, in the presence of a low interstitial fluid volume, which is a defect of the microcirculation. Defective proteins were shown to impair the amount of information in brain biomolecular mechanisms, with consequent neuronal and synaptic damage.

## 1. Introduction

The regulation of water balance is governed by a feedback mechanism involving the function and interaction of different regions of the central nervous system and the kidneys [1,2,3]. Plasma osmolality indicates the level of body hydration and hypothalamus osmoreceptors detect and are sensitive to its variations [4]. High plasma osmolality, increased to levels above a physiologic threshold (290 to 295 mOsm per kilogram of water), leads to secretion of the peptide hormone, vasopressin, from the vasopressinergic nerve endings in the neurohypophysis. Vasopressin binds receptors in the kidney that decrease the excretion of water, and subsequently, a greater fraction of filtered water is returned to the blood. This process lowers the plasma osmolality, reduces the stimulus for vasopressin secretion and thirst and completes the feedback loop [5]. Failure of this mechanism, which is commonly observed in hospitalized patients, results in a variety of water balance disorders. More recent evidence has clarified that the aging process is commonly related to multiple abnormalities in water homeostasis, highlighting its effects on morbidity, cognition, osteoporosis, fractures, gait instability, and mortality [4]. In older patients, water homeostasis regulation can be altered because of multifactorial mechanisms, such as renal function alterations, body composition and hypothalamic–pituitary regulation of thirst and vasopressin secretion changes [4]. Several consequences of these changes can appear, such as homeostatic inelasticity [6], that is, a limited ability to adjust water balance, leading to dehydration and hyperosmolality [4,7,8]. This defect could be a consequence to the loss of normal neural pathways that transmit sensory input to higher cortical centers where stimuli are perceived, and from which the response emanates [4,9]. We consider, in accordance with the recent hydromolecular hypothesis [10], the abnormalities of water homeostasis to be early expressions of neuronal dysfunction, brain atrophy, chronic cerebrovasculopathy and Alzheimer’s disease. In light of this assumption, the aim of the work is to evaluate and study the relationships between cognitive impairment, behavioral disorders and impaired serum calculated osmolality.

## 2. Materials and Methods

The present study was conducted according to the Declaration of Helsinki, the Guidelines for Good Clinical Practice and the Guidelines for Strengthening the Reporting of Observational Studies in Epidemiology, and it was approved by the local ethics committee for human experimentation (Prot. No. 3877/DS). The study was an observational study, in which the assignment of an intervention to the participants, its effect assessment and health-related biomedical or behavioural outcomes are not considered. In the present study, healthy participants were recruited as control subjects.

### 2.1. Study Sample

From January 2015 to March 2017, we screened older subjects who had attended the Cognitive Impairment Evaluation Unit of the Complex Unit of Geriatrics of Istituto di Ricovero e Cura a Carattere Scientifico (IRCCS) “Casa Sollievo della Sofferenza” for possible study enrollment. After hospitalization, these subjects were visited as outpatients. The control group patients were recruited in the Geriatric Unit and were categorized as having no cognitive impairment by a clinical, cognitive, affective and functional assessment. We obtained written, informed consent for research from each patient, or from relatives or a legal guardian. All subjects were Caucasian, with no individuals of Jewish, Eastern European or North African descent, with most individuals having Southern Italian ancestry, and having lived in Southern Italy for at least three generations.

Inclusion criteria were as follows: (1) age ≥65 years; (2) diagnosis of a subjective cognitive impairment (SCI) according to the Subjective Cognitive Decline Initiative (SCD-I) Working Group [10]; (3) diagnosis of late-life depression (LLD) according to the (Diagnostic and Statistical Manual of Mental Disorders Fifth Edition) DSM 5 [11] criteria; (4) diagnosis of mild cognitive impairment (MCI) according to the National Institute on Aging—Alzheimer’s Association (NIAAA) [12] criteria; (5) diagnosis of Alzheimer’s disease (AD) according to the NIAAA [13] criteria; (6) diagnosis of Lewy body disease (LBD) according to the (Dementia with Lewy bodies) DLB consortium [14] criteria; (7) diagnosis of vascular dementia (VaD) according to the criteria of the National Institute of Neurological Disorders and Stroke—Association Internationale pour la Recherche et l’Enseignement en Neurosciences (NINDS-AIREN) [15] work group; (8) the ability to provide informed consent or the availability of a relative or legal guardian in the case of severe demented patients. The exclusion criteria were as follows: (1) diagnosis of mixed dementia (MxD); (2) presence of a serious comorbidity, tumors, other diseases or physiological status (ascertained blood infections, vitamin B12 deficiency, anemia, disorders of the thyroid, kidneys, or liver), that could be causally related to cognitive impairment; (3) history of alcohol or drug abuse, head trauma, and other causes that could cause memory impairment. 

In the control group, we included older patients consecutively evaluated in the same centre who had no cognitive impairment or depression symptoms.

### 2.2. Clinical, Cognitive, Affective and Functional Assessment

The medical statuses of the patients were collected by a structured interview, a clinical evaluation and a review of records from the patients’ general practitioners. 

In all patients, cognitive status was defined with the Mini-Mental State Examination (MMSE) [16] and the Clinical Dementia Rating Scale (CDR) [17] after a brief interview with the caregiver. The diagnosis of dementia was always supported by neuroimaging evidences (computed tomography scan and/or nuclear magnetic resonance). A differential diagnosis between AD and VaD was also carried out, based on the Hachinski Ischemic Score (HIS), to address unclear AD/VaD diagnoses [18]. In particular, scores ≤ 4 were considered as probable AD and scores ≥ 7 were included into the VaD group. Scores between 5 and 6 were diagnosed as mixed dementia (MxD) and were excluded from the study. 

The affective status was assessed using the 21-item version of the Hamilton Depression Rating Scale (HDRS-21) [19]. Diagnosis of LLD was made according to the DSM 5 criteria.

In all patients, functional status was assessed by the Activities of Daily Living (ADL) [20] and Instrumental Activities of Daily Living (IADL) [21] scales, nutritional status was explored with the Mini Nutritional Assessment (MNA) [22], the risk of developing pressure sores was evaluated by the Exton-Smith Scale (ESS) [23], and comorbidity was examined using the Cumulative Illness Rating Scale Comorbidity Index (CIRS-CI) [24].

### 2.3. Value Quantification of Serum Osmolality

Serum osmolality is an accurate indicator of hydration status in older adults. Glucose, urea, and electrolyte concentrations [sodium (Na^+^), potassium (K^+^), and chloride (Cl^−^) are used to calculate an indirect estimate of serum osmolality. In order to obtain a reliable indicator of serum osmolality, we used the equation for calculated serum osmolality developed by Khajuria and Krahn [25] [1.86 (Na^+^ + K^+^) + (1.15 × glucose) + urea + 14], where all components were measured in mmol/L. The equation we adopted has shown accuracy in frail older people, with and without diabetes [26]. Participants were categorized as (1) being normally hydrated (calculated serum osmolality from 275 to 295 mmol/kg); (2) having impending dehydration (calculated serum osmolality from 295 to 300 mmol/kg); or (3) having current dehydration (calculated serum osmolality 300 mmol/kg). Kidney function was calculated with the CKD-EPI equation: estimated glomerular filtrate rate (eGFR) = 141 × min(Scr/κ,1)^α^ × max(Scr/κ, 1)^−1.209^ × 0.993^Age^ × 1.018 (if female) × 1.159 (if black) [27], where Scr is serum creatinine (mg/dL), κ is 0.7 for females and 0.9 for males, α is −0.329 for females and −0.411 for males, min indicates the minimum value of Scr/κ or 1, and max indicates the maximum value of Scr/κ or 1 [28]. eGFR stages were defined as >90 (healthy kidneys or kidney damage with normal or high eGFR), 60–89 (kidney damage and mild decrease in eGFR), 30–59 (moderate decrease in eGFR), 15–29 (severe decrease in eGFR), and <15 mL/min/1.73 m^2^ (kidney failure) [28].

### 2.4. Statistical Analyses

For dichotomous variables, hypotheses regarding differences between groups were tested using the Fisher’s exact test. This analysis was made using the 2-Way Contingency Table Analysis. For continuous variables, normal distribution was verified by the Shapiro–Wilk normality test and the one-sample Kolgomorov–Smirnov test. For normally-distributed variables, hypotheses regarding differences among the groups were compared by means of the Welch two sample *t*-test or by means of the analysis of variance (ANOVA) using the general linear model. For non-normally distributed variables, hypotheses regarding differences among the groups were compared by means of the Wilcoxon rank sum test with continuity correction or by means of the Kruskal–Wallis rank sum test. Risks (adjusted by age) are reported as odds ratios (OR) along with their 95% confidence interval (CI). All the statistical analyses were made with the R Ver. 2.8.1 statistical software package (The R Project for Statistical Computing; available at URL http://www.r-project.org/). Tests in which the *p*-value was smaller than the type I error rate, α = 0.05, were declared significant.

## 3. Results

During the enrolment period, 1135 older patients were screened for inclusion in the study. Of these, nine patients were excluded because they were younger than 65 years, 12 patients had an incomplete examination and 23 patients had MxD. Thus, the final population included 1091 patients, 542 men (49.70%) and 549 women (50.3%), with a mean age of 78.80 ± 6.72 years and an age range from 65 to 97 years. Therefore, 520 patients (Male: 326, Female: 194; mean age of 77.64 ± 6.60 years, range 65–95 years) were included in the control group, and 571 patients (Male: 216, Female: 355; mean age of 79.94 ± 6.67 years, range 65–97 years) had cognitive decline and/or depression mood (CD-DM). The demographic and clinical characteristics of the control group and patients with CD-DM are summarized in Table 1. The patients with CD-DM were less likely to be male (37.80% vs. 62.70%, *p* < 0.001), and more likely to be older (79.94 vs. 77.64 years, *p* < 0.001), have clear significant cognitive impairment (MMSE: 18.00 vs. 29.00, *p* < 0.001), and show the presence of a depressed mood (HDRS-21: 10.96 vs. 2.48, *p* < 0.001) compared to individuals in the control group. CD-DM patients showed a major impairment in ADL (4.14 vs. 6.00, *p* < 0.001), IADL (3.31 vs. 8.00, *p* < 0.001), MNA (23.02 vs. 24.26, *p* < 0.001), and ESS (17.32 vs. 19.32, *p* < 0.001) compared to individuals in the control group. The two groups did not differ in CIRS scores (*p* = 0.241), or glucose (*p* = 0.903), urea (*p* = 0.571), and Scr (*p* = 0.107) values. The CD-DM patients had a higher electrolyte concentration (Na^+^: 141.00 vs. 140.28 mmol/L, *p* < 0.001; K^+^: 4.52 vs. 4.27 mmol/L, *p* < 0.001; Cl^−^: 105.04 vs. 103.73 mmol/L, *p* < 0.001), risk of dehydration (osmolality: 298.44 vs. 296.53 mmol/kg, *p* < 0.001), and level of kidney damage (eGFR mean score: 69.64 vs. 72.21 mL/min/1.73 m2, *p* = 0.021) than the control group. 

As explained in Table 2, the patients affected to CD-DM were collected in six groups: (1) 27 patients with SCI, (2) 125 patients with LLD and cognitive impairment, (3) 78 patients with MCI, (4) 122 patients with AD, (5) 203 patients with VaD, and (6) 16 patients with LBD. 

The patients with MCI included more males (SCI: 40.70% vs. LLD with cognitive impairment: 20.00% vs. MCI: 53.80% vs. AD: 42.60% vs. VaD: 39.00% vs. LBD: 43.80%) than other groups of patients. AD, VaD and LBD patients included more older individuals (SCI: 77.33 vs. LLD with cognitive impairment: 77.75 vs. MCI: 78.10 vs. AD: 79.00 vs. VaD: 83.00 vs. LBD: 79.13 years), had higher cognitive impairment based on the MMSE mean score (SCI: 27.64 vs. LLD with cognitive impairment: 19.92 vs. MCI: 25.14 vs. AD: 13.99 vs. VaD: 15.24 vs. LBD: 17.72), had higher functional impairment based on the ADL (SCI: 6.00 vs. LLD with cognitive impairment: 4.45 vs. MCI: 6.00 vs. AD: 3.57 vs. VaD: 3.36 vs. LBD: 3.56) and IADL mean scores (SCI: 8.00 vs. LLD with cognitive impairment: 3.82 vs. MCI: 8.00 vs. AD: 1.60 vs. VaD: 1.71 vs. LBD: 2.06), had greater risk of malnutrition based on the MNA mean score (SCI: 27.03 vs. LLD with cognitive impairment: 23.26 vs. MCI: 26.00 vs. AD: 21.96 vs. VaD: 21.90 vs. LBD: 22.63), and had risk of developing pressure sores based on the ESS mean score (SCI: 19.00 vs. LLD with cognitive impairment: 17.66 vs. MCI: 18.88 vs. AD: 17.21 vs. VaD: 16.32 vs. LBD: 17.63) than other groups.

Patients with LLD and cognitive impairment had higher mean scores in the HRSD-21 assessment (SCI: 3.30 vs. LLD with cognitive impairment: 14.66 vs. MCI: 4.91 vs. AD: 10.11 vs. VaD: 13.09 vs. LBD: 12.23) than other groups. 

VaD patients showed more comorbidities based on their CIRS mean scores (SCI: 2.00 vs. LLD with cognitive impairment: 2.42 vs. MCI: 1.92 vs. AD: 1.95 vs. VaD: 2.72 vs. LBD: 2.13). 

AD and VaD patients had higher mean scores for glucose (SCI: 5.30 vs. LLD with cognitive impairment: 5.30 vs. MCI: 5.30 vs. AD: 5.50 vs. VaD: 5.80 vs. LBD: 4.60) and Scr (SCI: 73.50 vs. LLD with cognitive impairment: 75.60 vs. MCI: 79.50 vs. AD: 79.30 vs. VaD: 87.90 vs. LBD: 71.3) content. Cognitively impaired patients with AD or LLD had higher Na^+^ concentrations (SCI: 140.70 vs. LLD with cognitive impairment: 141.38 vs. MCI: 140.68 vs. AD: 141.80 vs. VaD: 140.42 vs. LBD: 141.19).

Patients with LLD and cognitive impairment and patients with VaD had higher mean in urea (SCI: 7.10 vs. LLD with cognitive impairment: 7.40 vs. MCI: 7.00 vs. AD: 6.80 vs. VaD: 8.20 vs. LBD: 6.60), and K^+^ (SCI: 4.49 vs. LLD with cognitive impairment: 4.51 vs. MCI: 4.44 vs. AD: 4.45 vs. VaD: 4.60 vs. LBD: 4.24) scores than other groups. 

SCI patients had higher Cl^−^ mean scores (SCI: 105.30 vs. LLD with cognitive impairment: 104.66 vs. MCI: 105.00 vs. AD: 105.24 vs. VaD: 105.02 vs. LBD: 105.06) than other groups.

Finally, cognitively impaired patients with VaD, AD and LLD had a higher risk of kidney damage based on eGFR scores (SCI: 76.21 vs. LLD with cognitive impairment: 72.39 vs. MCI: 73.17 vs. AD: 71.82 vs. VaD: 63.84 vs. LBD: 76.92), as well as a higher dehydration risk (SCI: 297.23 vs. LLD with cognitive impairment: 298.77 vs. MCI: 297.00 vs. AD: 299.23 vs. VaD: 298.63 vs. LBD: 296.36) compared with the other groups of patients.

**Figure 1:**
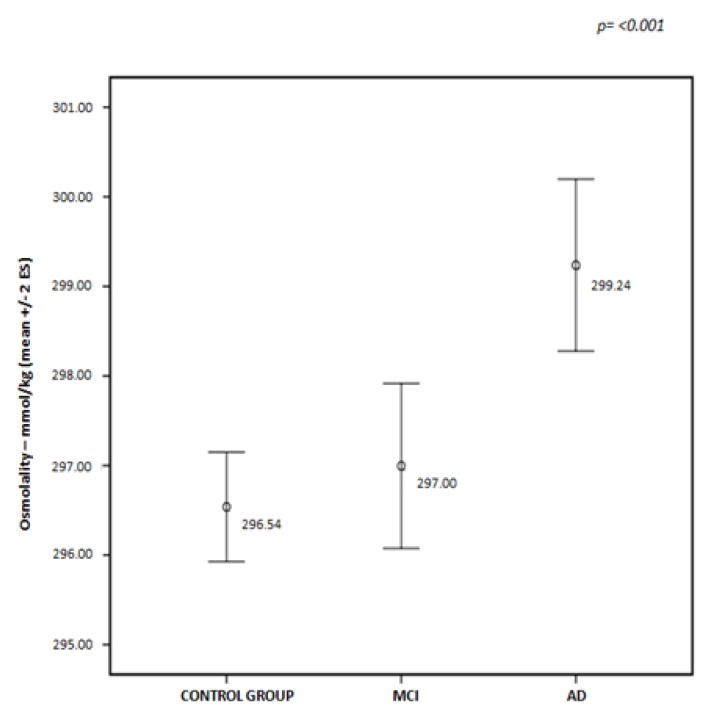
Distribution of osmolality mean values in control group, and mild cognitive impairment (MCI) and Alzheimer’s disease (AD) patients.

As shown in Figure 1, a significant difference was observed between the values of osmolality calculated for AD patients compared with the control group and MCI patients; AD patients showed a major risk for current dehydration (*p* ≤ 0.001).

As shown in Table 3, dehydration was associated with the risk of developing dementia conditions, like AD or VaD (OR = 2.016, *p* < 0.001).

**Figure 2:**
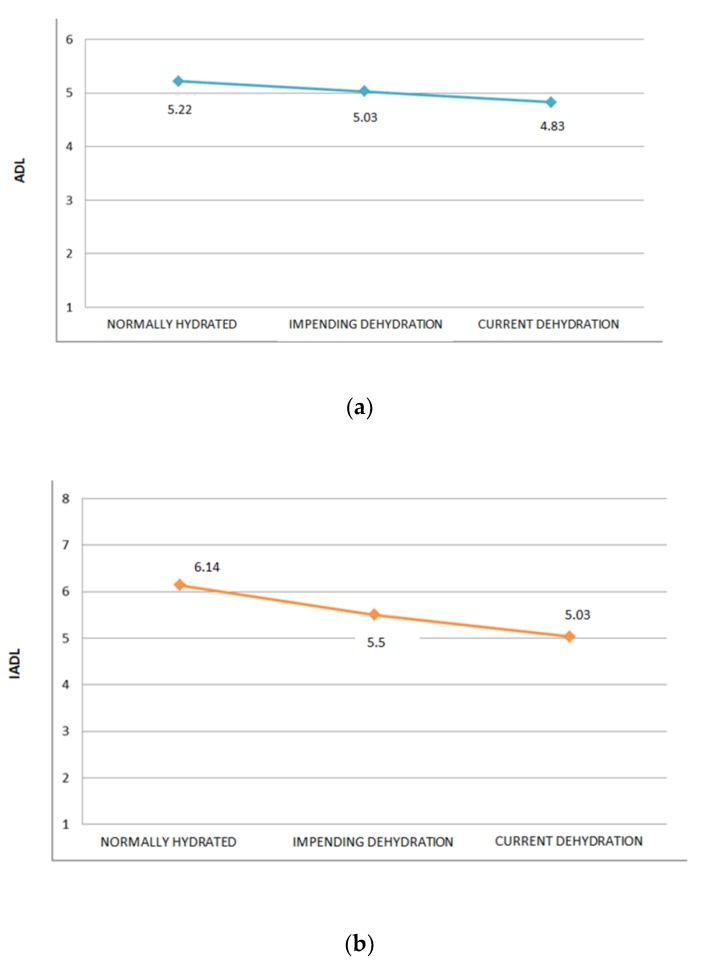

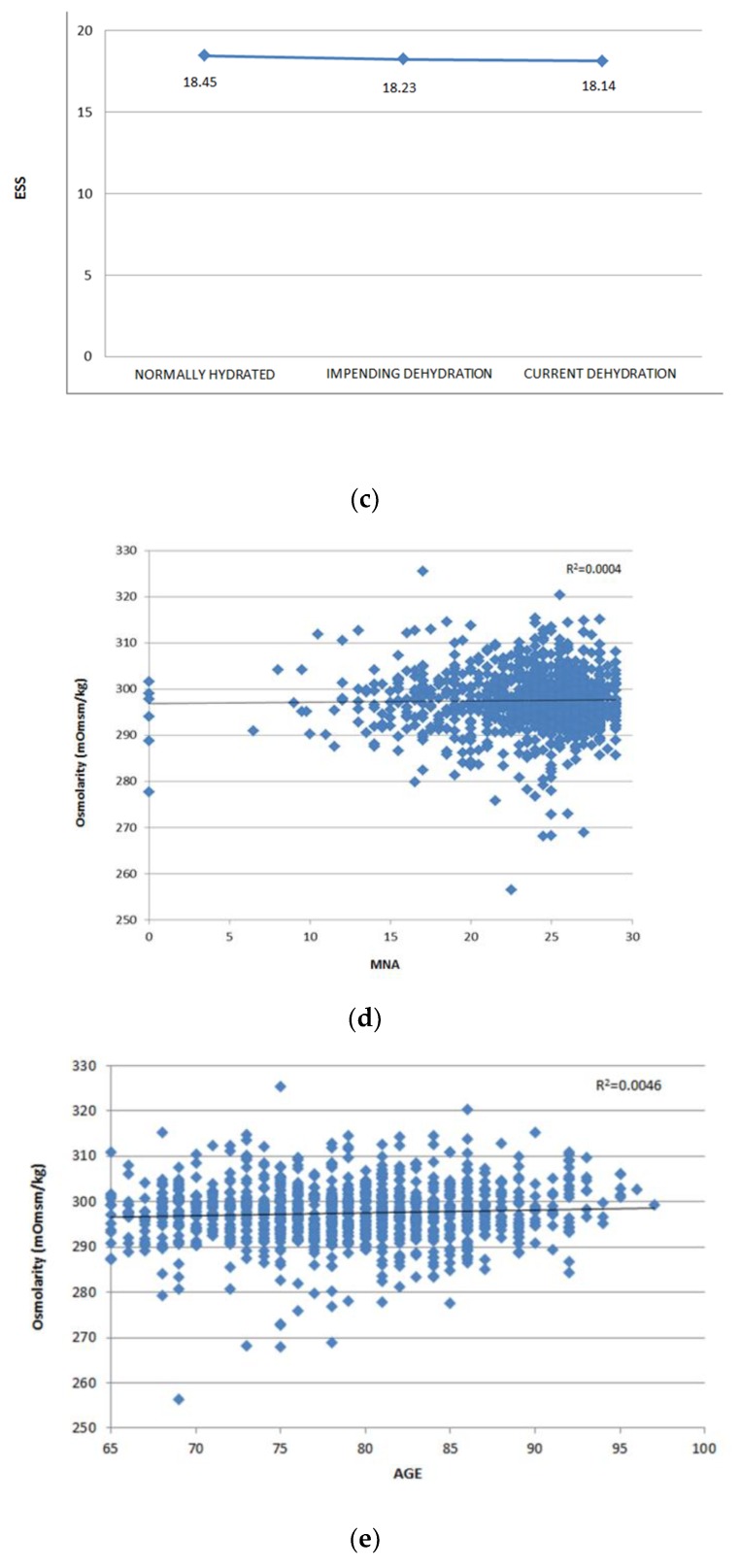
(**a**) Multivariate analysis of dehydration risk according to Activities of Daily Living (ADL) scores; (**b**) multivariate analysis of dehydration risk according to Instrumental Activities of Daily Living (IADL) scores; (**c**) multivariate analysis of dehydration risk according to Exton-Smith Scale (ESS) scores; (**d**) linear regression model of dehydration risk according to age of patients; (**e**) linear regression model of dehydration risk according to Mini Nutritional Assessment (MNA) scores.

As shown in Figure 2a,b, the presence of dehydration was associated with ADL (*p* ≤ 0.001) and IADL (*p* ≤ 0.001) scores, but was independently associated with the ESS (*p* = 0.54), as shown in Figure 2c. Moreover, the dehydration risk was independently associated with age (*r*^2^ = 0.0046, *p* = 0.77), and MNA (*r*^2^ = 0.0004, *p* = 0.48), as shown in Figure 2d,e, respectively.

**Figure 3:**
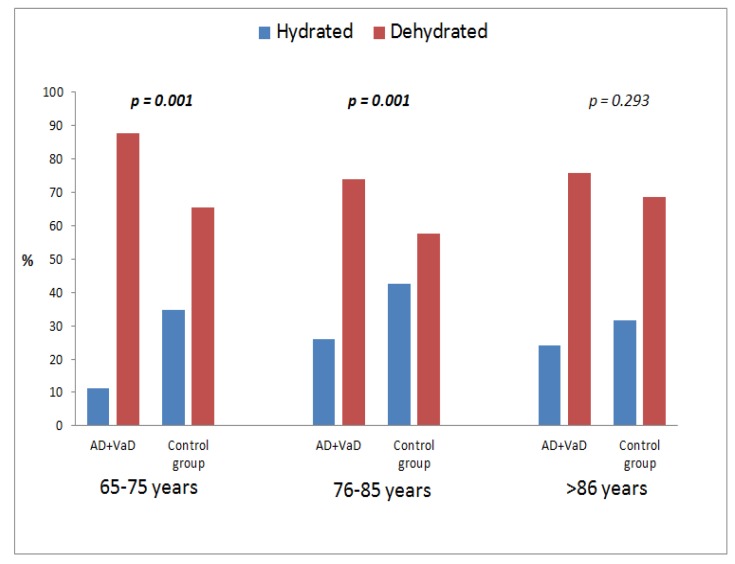
Distribution of dementia (AD and VaD) patients and control group according to dehydration risk and age.

In Figure 3, using the age ranges of patients, it was shown that younger patients (65–75 and 76–85 years) with dementia (AD and VaD) were significantly more dehydrated than patients without dementia (65–75 years, *p* = 0.001; 76–85 years, *p* = 0.001; ≥86 years, *p* = 0.293).

## 4. Discussion

Dehydration has been reported to be the most common fluid and electrolyte imbalance in older adults [29,30,31]. Recent clinical studies have shown that the hydration state affects cognitive performance, particularly visual attention and mood [32]. The changes in extracellular osmolality inevitably affect the intracellular environment determining important alterations in the volume and function of cellular mechanisms causing irreversible morphological and functional damage. Thus, these changes could be major contributing factors to age-related neurovascular vulnerabilities and are currently under intense investigation as potential therapeutic targets. It has been hypothesized that chronic hypovolemia, due to hypohydration, is perhaps one of the principal mechanisms behind the development of obesity, diabetes, hypertension, and even Alzheimer’s disease [33,34,35,36]. Hospitalized older adults suffering from dehydration have been reported to have mortality rates as high as 45–46% [31,37,38], but only a few studies exist regarding water intake and excretion in older patients. Disability, visual impairment, speaking ability, incontinence and number of ingestion sessions could be possible risk factors for a decreased fluid intake but the results obtained on this topic are few and contradictory [39,40,41,42,43]. The majority of medications used to treat cardiovascular disease block the renin–angiotensin–aldosterone system, yet this system is activated physiologically by hypovolemia [42]. Most significantly, dehydration has been associated with increased mortality rates among hospitalized older adults [2]. This “hypothesis” of dehydration is supported further by studies showing that total body water decreases with age [44] as well as with increasing Body Mass Index [37], thus suggesting that aged and/or obese and/or diabetic patients could be chronically dehydrated. Then, hypovolemia, resulting from systemic dehydration, has an important negative effect on cell metabolism [45,46] which could also reduce brain volume [47,48] cause impairment of cerebral microcirculation, alter synaptic morphology, and cause progressive loss of synapses and glial activation, which are characteristic hallmarks of aging and predispose older patients to develop cognitive impairment. Our work shows an impairment of calculated serum osmolality in MCI, VaD and AD patients. As evidenced by the results, this data is not a consequence of the functional and nutritional state. Moreover, in the current study, younger patients (65–75 and 76–85 years) with dementia (AD and VaD) were significantly more dehydrated than patients without dementia. 

In Accordance with our outcomes, all patient groups with CD-DM showed an increased prevalence of dehydration when compared to the control group, even if analysed by age range. In particular, when AD patients and MCI patients were compared (considering that these can develop AD in future), we observed a major serum hyperosmolality risk. Moreover, more severe cognitive impairment associated with dehydration were observed in AD and VaD patients. The central nervous system, in spite of being a highly lipophilic organ, consists of 80% water, stored principally in astrocytes that respond to peripheral dehydration by upregulation of AQP-4 proteins on their end-feet processes, probably to preserve water [48]. The hydromolecular hypothesis intends to explain the relationship between dehydration and cognitive impairment in older patients as resulting from protein misfolding and aggregation in the presence of low interstitial fluid volume which is a defect of the microcirculation. Defective proteins impair the amount of information in brain biomolecular mechanisms with consequent neuronal and synaptic damage. Our conclusions suggest continuing to deepen the biology and physiopathology of the topics discussed and considering the importance of assessing and treating disorders of fluid balance, a critical step when dealing with the older patients and disorders related to the aging brain. In light of our outcomes, further studies are needed to define clearly if dehydration is a cause or effect of the onset of dementia.

## Figures and Tables

**Table 1:** Demographic and clinical characteristics of control group and patients with cognitive decline and/or depression mood (CD-DM).

	Control Group *n* = 520	CD-DM *n* = 571	*p* Value	Whole Population *n* = 1091
Male sex *	326 (62.7%)	216 (37.8%)	<0.001	542 (49.7%)
Age (years) **	77.64 ± 6.60 (65–95)	79.94 ± 6.67 (65–97)	<0.001	78.80 ± 6.72 (65–97)
MMSE **	29.00 ± 0.61 (29–30)	18.00 ± 6.72 (0–30)	<0.001	23.28 ± 7.32 (0–30)
HRSD-21 **	2.48 ± 1.61 (1–6)	10.96 ± 6.93 (0–31)	<0.001	6.57 ± 6.66 (0–31)
ADL **	6.00 ± 0.00 (6–6)	4.14 ± 1.88 (0–6)	<0.001	4.98 ± 1.65 (0–6)
IADL **	8.00 ± 0.00 (8–8)	3.31 ± 3.18 (0–8)	<0.001	5.39 ± 3.38 (0–8)
MNA **	24.26 ± 3.42 (9–29)	23.02 ± 4.45 (0–29)	<0.001	23.61 ± 4.00 (0–29)
ESS **	19.32 ± 0.78 (18–20)	17.32 ± 2.35 (9–20)	<0.001	18.27 ± 2.05 (9–20)
CIRS **	2.23 ± 1.39 (0–7)	2.33 ± 1.50 (0–9)	0.241	2.28 ± 1.45 (0–9)
Glucose (mmol/L) **	5.53 ± 1.96 (2.24–17.22)	5.54 ± 1.51 (3.64–14.42)	0.903	5.54 ± 1.76 (2.23–17.22)
Urea (mmol/L) **	7.44 ± 2.61 (2.34–19.00)	7.55 ± 3.12 (2.82–34.53)	0.571	7.43 ± 2.82 (2.31–34.53)
Scr (µmol/L) **	83.82 ± 27.95 (35.42–221)	81 ± 27.22 (36.21–212.20)	0.107	82.40 ± 27.63 (35.42–221)
Na^+^ (mmol/L) **	140.28 ± 3.47 (120–151)	141.00 ± 2.46 (133–151)	<0.001	140.65 ± 3.00 (120–151)
K^+^ (mmol/L) **	4.27 ± 0.49 (2.80–6.46)	4.52 ± 0.46 (3.31–7.78)	<0.001	4.40 ± 0.50 (2.80–7.78)
Cl^−^ (mmol/L) **	103.73 ± 4.48 (85–142)	105.04 ± 3.08 (86–116)	<0.001	104.40 ± 3.86 (85–142)
eGFR (mL/min/1.73 m^2^) **	72.21 ± 18.03 (19.52–107.91)	69.64 ± 18.57 (16.66–106.50)	0.021	71.00 ± 18.35 (16.66–107.91)
Osmolality (mmol/kg) **	296.53 ± 6.98 (256–315)	298.44 ± 5.53 (283–325)	<0.001	297.53 ± 6.33 (256–325)

* Absolute frequency (percentage). ** Mean ± SD (range). MMSE, Mini Mental State Examination; HRSD-21, Hamilton Rating Scale for Depression; ADL, Activities of Daily Living; IADL, Instrumental Activities of Daily Living; MNA, Mini Nutritional Assessment; ESS, Exton-Smith Scale; CIRS, Cumulative Illness Rating Scale; Scr, serum creatinine; Na^+^, sodium; K^+^, potassium; Cl^−^, chloride; eGFR, estimated glomerular filtration rate.

**Table 2:** Clinical features of patients affected with cognitive decline and/or depression mood (CD-DM).

	SCI *n* = 27	LLD with Cognitive Impairment *n* = 125	MCI *n* = 78	AD *n* = 122	VaD *n* = 203	LBD *n* = 16
Male sex *	11 (40.70%)	25 (20.00%)	42 (53.80%)	52 (42.60%)	79 (39.00%)	7 (43.80%)
Age (years) **	77.33 ± 6.81 (66–92)	77.75 ± 6.50 (65–93)	78.10 ± 6.86 (65–97)	79.00 ± 6.31 (65–92)	83.00 ± 6.00 (65–96)	79.13 ± 5.32 (69–91)
MMSE **	27.64 ± 1.93 (24–30)	19.92 ± 5.44 (0–29)	25.14 ± 1.49 (24–28)	13.99 ± 5.81 (0–27)	15.24 ± 5.87 (0–29)	17.72 ± 4.68 (9–24)
HRSD-21 **	3.30 ± 2.35 (0–7)	14.66 ± 5.60 (8–30)	4.91 ± 2.44 (0–7)	10.11 ± 6.52 (0–26)	13.09 ± 6.85 (0–31)	12.23 ± 9.19 (0–31)
ADL **	6.00 ± 0 (6–6)	4.45 ± 1.68 (0–6)	6.00 ± 0 (6–6)	3.57 ± 1.88 (0–6)	3.36 ± 1.80 (0–6)	3.56 ± 1.75 (1–6)
IADL **	8.00 ± 0 (8–8)	3.82 ± 3.15 (0–8)	8.00 ± 0 (8–8)	1.60 ± 2.34 (0–8)	1.71 ± 2.49 (0–8)	2.06 ± 2.83 (0–8)
MNA **	27.03 ± 1.79 (23–29)	23.26 ± 3.95 (9–29)	26.00 ± 3.10 (13–29)	21.93 ± 4.96 (0–29)	21.90 ± 4.39 (6–28)	22.63 ± 3.35 (16–28)
ESS **	19.00 ± 1.44 (14–20)	17.66 ± 2.12 (12–20)	18.88 ± 1.44 (15–20)	17.21 ± 2.29 (9–20)	16.32 ± 2.46 (9–20)	17.63 ± 1.71 (14–20)
CIRS **	2.00 ± 1.17 (0–4)	2.42 ± 1.43 (0–6)	1.92 ± 1.54 (0–6)	1.95 ± 1.28 (0–6)	2.72 ± 1.60 (0–9)	2.13 ± 1.50 (0–5)
Glucose (mmol/L) **	5.32 ± 1.33 (4.00–8.30)	5.29 ± 1.20 (3.85–10.44)	5.35 ± 1.50 (3.93–11.72)	5.57 ± 1.54 (3.62–14.43)	5.80 ± 1.81 (3.72–12.22)	4.67 ± 0.75 (3.63–6.55)
Urea (mmol/L) **	7.14 ± 2.35 (4.25–14.00)	7.40 ± 3.41 (3.52–34.55)	7 ± 2.02 (4.00–16.32)	6.82 ± 2.45 (2.84–17.33)	8.20 ± 3.61 (2.85–28.34)	6.63 ± 1.75 (4.72–9.73)
Scr (µmol/L) **	73.53 ± 20.81 (39.82–152.00)	75.60 ± 25.51 (36.22–198.92)	79.55 ± 22.47 (38.00–168)	79.36 ± 26.78 (38.00–160.92)	87.93 ± 30 (38.93–212.24)	71.33 ± 21.42 (46.84–120.26)
Na^+^ (mmol/L) **	140.70 ± 2.31 (137–148)	141.38 ± 2.84 (135–151)	140.68 ± 2.17 (134–145)	141.80 ± 2.40 (134–147)	140.42 ± 2.24 (133–147)	141.19 ± 2.13 (137–144)
K^+^ (mmol/L) **	4.49 ± 0.35 (3.97–5.20)	4.51 ± 0.47 (3.50–6.00)	4.44 ± 0.39 (3.70–5.62)	4.45 ± 0.38 (3.31–5.58)	4.60 ± 0.53 (3.44–7.78)	4.24 ± 0.36 (3.55–4.70)
Cl^−^ (mmol/L) **	105.30 ± 2.49 (101–110)	104.66 ± 2.94 (98–112)	105.00 ± 2.28 (100–109)	105.24 ± 3.36 (86–112)	105.02 ± 3.33 (96–116)	105.06 ± 3.17 (97–112)
eGFR (mL/min/1.73 m^2^) **	76.21 ± 15.65 (36.22–100.52)	72.39 ± 17.68 (20.72–106.50)	73.17 ± 16.10 (30.79–97.73)	71.82 ± 18.52 (24.26–106.24)	63.84 ± 19.27 (16.66–100.47)	76.92 ± 15.28 (52.21–95.16)
Osmolality (mmol/kg) **	297.23 ± 4.43 (288.00–308.00)	298.77 ± 6.44 (285.00–325.50)	297.00 ± 4.05 (285.00–306.00)	299.23 ± 5.30 (286.50–312.40)	298.63 ± 5.66 (283.00–314.50)	296.36 ± 4.23 (289.10–302.60)

* Absolute frequency (percentage). ** Mean ± SD (range). SCI, subjective cognitive impairment; LLD, late-life depression; MCI, Mmild cognitive impairment; AD, Alzheimer’s disease; VaD, vascular dementia; LBD, Lewy body disease; MMSE, Mini Mental State Examination; HRSD-21, Hamilton Rating Scale for Depression; ADL, Activities of Daily Living; IADL, Instrumental Activities of Daily Living; MNA, Mini Nutritional Assessment; ESS, Exton Smith Scale; CIRS, Cumulative Illness Rating Scale; Scr, serum creatinine; Na^+^, sodium; K^+^, potassium; Cl^−^, chloride; eGFR, estimated glomerular filtration rate.

**Table 3:** Logistic regression analysis of dehydration risk in control group and patients with Alzheimer’s disease (AD) and vascular dementia (VaD).

	Control Group *n* = 520	AD + VaD *n* = 325	OR	95% CI	*p*-Value
Hydrated *	196 (37.7%)	75 (23.1%)	2.016	1.474–2.758	<0.001
Dehydrated *	324 (62.3%)	250 (76.9%)			

* Absolute frequency (percentage).
